# Robust 3D image reconstruction of pancreatic cancer tumors from histopathological images with different stains and its quantitative performance evaluation

**DOI:** 10.1007/s11548-019-02019-8

**Published:** 2019-07-02

**Authors:** Mauricio Kugler, Yushi Goto, Yuki Tamura, Naoki Kawamura, Hirokazu Kobayashi, Tatsuya Yokota, Chika Iwamoto, Kenoki Ohuchida, Makoto Hashizume, Akinobu Shimizu, Hidekata Hontani

**Affiliations:** 1 Nagoya Institute of Technology, Gokiso-cho, Showa-ku, Nagoya, Japan; 2 Kyushu University, 3-1-1 Maidaishi, Higashi-ku, Fukuoka, Japan; 3 Tokyo University of Agriculture and Technology, 2-24-16 Naka-cho, Koganei-shi, Tokyo Japan

**Keywords:** Image registration, 3D reconstruction, Histological sections, Multiple stains, Artifact detection

## Abstract

**Purpose:**

Histopathological imaging is widely used for the analysis and diagnosis of multiple diseases. Several methods have been proposed for the 3D reconstruction of pathological images, captured from thin sections of a given specimen, which get nonlinearly deformed due to the preparation process. The majority of the available methods for registering such images use the degree of matching of adjacent images as the criteria for registration, which can result in unnatural deformations of the anatomical structures. Moreover, most methods assume that the same staining is used for all images, when in fact multiple staining is usually applied in order to enhance different structures in the images.

**Methods:**

This paper proposes a non-rigid 3D reconstruction method based on the assumption that internal structures on the original tissue must be smooth and continuous. Landmarks are detected along anatomical structures using template matching based on normalized cross-correlation (NCC), forming jagged shape trajectories that traverse several slices. The registration process smooths out these trajectories and deforms the images accordingly. Artifacts are automatically handled by using the confidence of the NCC in order to reject unreliable landmarks.

**Results:**

The proposed method was applied to a large series of histological sections from the pancreas of a KPC mouse. Some portions were dyed primarily with HE stain, while others were dyed alternately with HE, CK19, MT and Ki67 stains. A new evaluation method is proposed to quantitatively evaluate the smoothness and isotropy of the obtained reconstructions, both for single and multiple staining.

**Conclusions:**

The experimental results show that the proposed method produces smooth and nearly isotropic 3D reconstructions of pathological images with either single or multiple stains. From these reconstructions, microanatomical structures enhanced by different stains can be simultaneously observed.

## Introduction

Microscopic images from histological sections are widely used for the analysis and definitive diagnosis of multiple diseases [8, 17]. It is one of the modalities with highest spatial resolution, allowing the observation of small anatomical structures at sub-micrometer scales. A common approach for obtaining a 3D microscopic image is to reconstruct it from a series of images of spatially continuous thin sections sliced from a target tissue, and several methods following this principle have been proposed [17, 21]. When mounted in a glass slide, each thin section gets non-rigidly deformed, requiring 3D reconstruction methods to properly register the original images in order to obtain a consistent reconstruction.

These registration methods can be roughly divided into two categories: intensity-based and landmark-based techniques [17], the latter being more memory efficient when processing very large pathological images. The methods in each of these categories can again be classified into two groups based on the criteria used to measure the quality of the registration. While some methods calculate the degree of match of two given images [2, 3, 16, 19], others measure the smoothness of the obtained 3D reconstruction [5, 7, 22]. The former approach tends to unnaturally deform three-dimensional structures into vertical straight shapes, due to the “banana” effect [15]. The method proposed in this paper performs the registration based on landmarks extracted from the original set of images, which are then deformed in order to produce a 3D image with smooth spatial patterns, under the assumption that the 3D patterns of the reconstructed image will be smooth when the corresponding landmarks themselves form smooth patterns.

The majority of the methods for reconstruction of 3D pathology images assume that the same staining is used for all available thin sections, usually Hematoxylin & Eosin (HE). However, it is uncommon to have only HE-stained sections, but instead multiple different stains, which allow a more complete understanding of the specimen under analysis [20]. The detection of corresponding landmarks is not straightforward when adjacent slices are dyed with different stains; nevertheless, this paper shows that simple template matching using normalized cross-correlation (NCC) can properly detect corresponding landmarks between several different combinations of staining. The level of confidence of the template matching is also used to automatically reject unreliable landmarks resulting from artifacts, such as folds and wrinkles, commonly present on pathological images of thin sections.

Several methods construct local feature descriptors that can explicitly label corresponding pixels from two differently stained images by unsupervised learning [4, 20]. These methods, however, can fail to assign similar labels when two local regions of similar color pattern in one image are stained differently in the other image, as the local feature descriptors cannot distinguish between the two regions in the former image. For instance, only some cells, which are difficult to distinguish in HE-stained images, will be marked brown in Ki67-stained images (active tumor cells). The most informative features for the correspondence of images with different stains are edge locations within the images. The NCC can evaluate the overlapping degree of these edges, and virtually all edges of one image can be observed in the adjacent images.

The accuracy of the reconstructed 3D image is quantitatively evaluated in the experiments. Many conventional criteria for accuracy evaluation cannot directly be applied to images reconstructed from slices of multiple stains. In this paper, the accuracy and isotropy of the reconstructed 3D image were evaluated by binarizing all slices of different stains and evaluating the complexity of contour shapes observed in the resulting images. This method demonstrates that the proposed method can reconstruct smooth and isotropic 3D images.

The contributions of this paper are threefold: (1) a method capable of reconstructing a 3D pathological image from a series of microscopic images of thin sections dyed with different stains is proposed, (2) a 3D pathological image of a pancreas tumor that is reconstructed from thin sections dyed with multiple stains is demonstrated, and (3) a method to quantitatively evaluate the smoothness and isotropy of reconstructed 3D images is proposed. Regarding (1), by employing the trajectories’ smoothness of corresponding landmarks as the criterion, the proposed method can simultaneously determine appropriate diffeomorphic mappings for each of the input slice images. As for (3), the method explicitly extracts boundaries of anatomical structures in order to evaluate their complexity, since color image patterns cannot be directly used for this evaluation when adjacent slices are dyed with different stains. This paper is an extension of work originally presented in MICCAI COMPAY 2018 [11]; the aforementioned contributions significantly expand over the previously published results.

**Fig. 1:**
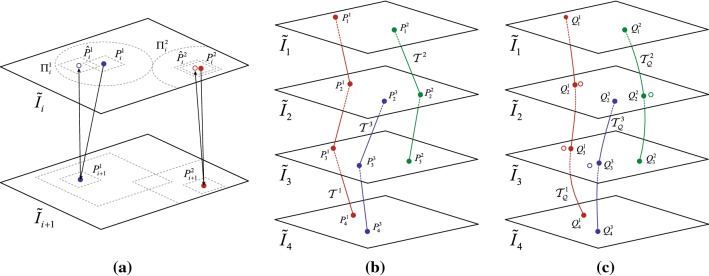
Landmark-based nonlinear registration: **a** detection of corresponding points by template matching, **b** trajectories ${{\mathcal {T}}^j}$ traversing images ${{{\tilde{I}}}_i}\left( {{\mathbf {x}}} \right)$ on landmarks $P_i^j$, and **c** smoothed trajectories ${\mathcal {T}}_{\mathcal {Q}}^j$ and the destination coordinates $Q_i^j$ [10]

## Image reconstruction

Let a set of *N* images corresponding to the thin slices of the original tissue be denoted as ${I_i}\left( {{\mathbf {u}}} \right)$, where ${{\mathbf {u}}} = {\left( {{u_1},{u_2}} \right) ^{\text {T}}}$ represents the coordinates in the original image and $i = 1 \ldots N$. The registration process begins by rigidly registering the images in order to remove large offsets and rotations [16], resulting in the set ${{{\tilde{I}}}_i}\left( {{\mathbf {x}}} \right) = {I_i}\left( {\rho _i^{ - 1} \circ {{\mathbf {u}}}} \right)$, where ${{\mathbf {x}}} = {\left( {{x_1},{x_2}} \right) ^{\text {T}}}$ are the coordinates on the new image set, with ${{\mathbf {x}}} = {\rho _i}\left( {{\mathbf {u}}} \right)$, and ${\rho _i}$ corresponds to the rigid transformation parameters.

The nonlinear deformation process starts from detecting a set of landmarks $P_{i=1}^j$ on the first image of the stack. Landmarks are randomly sampled at locations with large gradients of intensity according to the following probability:

1 $$\begin{aligned} p_i^j\left( {{\mathbf {x}}} \right) {=} \left\{ { \begin{array}{ll} {\left\| {\nabla {{{\tilde{I}}}_i}\left( {{\mathbf {x}}} \right) } \right\| /Z,} &{} {{\text {if}}} {\left( {\left\| {\nabla {{{\tilde{I}}}_i}\left( {{\mathbf {x}}} \right) } \right\| {>} T} \right) {\wedge } \left( {{{\mathbf {x}}} \in {\varPi } _i^j} \right) } \\ {0,} &{} {{\text {otherwise}}} \\ \end{array} } \right. \nonumber \\ \end{aligned}$$

where *Z* is a normalization factor and *T* is a threshold. The parameter ${{\varPi } _i^j}$ is used to define an area in which a landmark can be sampled, thus ensuring an uniform distribution of the landmarks and avoiding oversampling. Specifically, after a landmark is found, no other landmarks will be sampled on the surrounding area. Although the sampling process does not actively follow any specific anatomical structure, the landmarks are likely to be generated along the edges such structures, as they correspond to the regions with large gradients. By setting the threshold *T* to a low value, only areas with very low contrast will be ignored, such as the empty areas of the slide around the target specimen, in which no landmarks are expected to be detected.

For each landmark $P_i^j$ from ${{{\tilde{I}}}_i}\left( {{\mathbf {x}}} \right)$, a corresponding point $P_{i + 1}^j$ is searched in ${{{\tilde{I}}}_{i+1}}\left( {{\mathbf {x}}} \right)$ using NCC-based template matching, as shown in Fig. 1a. The NCC uses a template size *D* and a maximal search distance of 2*D* from the original coordinate of $P_i^j$. The process is repeated over all *N* images, creating a series of polygonal trajectories ${{\mathcal {T}}^j} = \overline{P_{{s^j}}^jP_{{s^j} + 1}^j \ldots P_{{t^j}}^j}$ formed by the sequence of corresponding points ${{\mathcal {P}}^j} = \left\{ {P_i^j\left| {i \in \left[ {{s^j},{t^j}} \right] } \right. } \right\}$, where ${{s^j}}$ and ${{t^j}}$ correspond to the first and last images traversed by the *j*th trajectory, with $1 \leqslant {s^j} < {t^j} \leqslant N$. Due to the aforementioned nonlinear deformations introduced on the images, these trajectories tend to be very jagged, as shown in Fig. 1b.

Several alternatives exist for detecting corresponding landmarks, among which mutual information [14] is widely used for registering images of different modalities, but its high computational cost makes it unsuitable for large image datasets [1, 13]. The NCC is not only computationally simpler but also robust in the case of template matching between images with different stains. The cross-correlation computed by the NCC is maximized when both inputs change synchronously. Thus, it will present higher values when the large gradient templates selected by Eq. (1) are matched. Simultaneously, the normalization cancels most of the intensity differences, which is the key for multiple stain registration.

It must be noticed that not all trajectories span over all *N* images. Due to artifacts on the images, several false matchings can occur. In order to prevent such invalid correspondences, not only the coordinates of the maximal NCC value, but also the confidence of the NCC itself is used to determine if $P_{i+1}^j$ is a valid corresponding point. If the NCC value is lower than a threshold ${\theta _C}$, the candidate corresponding point will be discarded. Moreover, by applying backward template matching [24] as shown in Fig. 1a, if the resulting point ${\hat{P}}_i^j$ is further from $P_i^j$ than a distance threshold ${\theta _D}$, the candidate landmark will also be discarded. In both cases, the trajectory ${{\mathcal {T}}^j}$ is interrupted at ${{{\tilde{I}}}_i}\left( {{\mathbf {x}}} \right)$ and a new trajectory ${{\mathcal {T}}^k}$ is re-initiated at image ${{{\tilde{I}}}_{i+1}}\left( {{\mathbf {x}}} \right)$ if a valid landmark can be found using Eq. (1) in the area defined by ${{\varPi } _{i+1}^k}$.

The next step consists on smoothing these trajectories under the assumption that the original anatomical structures were smooth and continuous before the slicing of the specimen. This is achieved by minimizing the total variation in the trajectories as follows:

2 $$\begin{aligned} \left\{ {{{\mathbf {y}}}_{{s^j}}^j, \ldots ,{{\mathbf {y}}}_{{t^j}}^j} \right\} {=} \mathop {\arg \min }\limits _{{{\tilde{\mathbf {y}}}}_{{s^j}}^j, \ldots ,{{\tilde{\mathbf {y}}}}_{{t^j}}^j} \left( {\sum \limits _{n = {s^j}}^{{t^j} - 1} {{{\left\| {{{\tilde{\mathbf {y}}}}_n^j {-} {{\tilde{\mathbf {y}}}}_{n + 1}^j} \right\| }^2} {+} \lambda } {{\left\| {{{\tilde{\mathbf {y}}}}_n^j {-} {{\mathbf {x}}}_n^j} \right\| }^2}} \right) \nonumber \\ \end{aligned}$$

where $\lambda$ is a trade-off parameter, ${{{\mathbf {x}}}_i^j}$ corresponds to the original landmark coordinates and ${{\mathbf {y}}}_i^j$ correspond to the destination coordinates where the *j*th smoothed trajectories traverse the *i*th image. These new coordinates define new locations $Q_i^j$ for the landmarks, which in turn define a new set ${\mathcal {T}}_{\mathcal {Q}}^j = \overline{Q_{{s^j}}^jQ_{{s^j} + 1}^j \ldots Q_{{t^j}}^j}$ of trajectories, as shown in Fig. 1c. The smoothness of the resulting trajectories can be controlled by $\lambda$: overlarge values would create almost vertical lines, while too small values would not properly eliminate the irregularities of the trajectory.

Finally, the dense deformation mappings ${\phi _i}$ required to warp each image are calculated by interpolating each coarse deformation field defined by the landmarks’ displacement using B-splines [12]. This method produces a diffeomorphic mapping ${{\mathbf {y}}}_i = {\phi _i} \circ {{\mathbf {x}}}_i$ with hard constrains [18]. A sufficiently dense set of landmarks, controlled by the parameter ${{\varPi } _i^j}$ in Eq. (1), guarantees that the final deformation field obtained after the B-spline interpolation will be smooth. As each field is independently interpolated, the B-spline calculations can be efficiently parallelized.

**Fig. 2:**
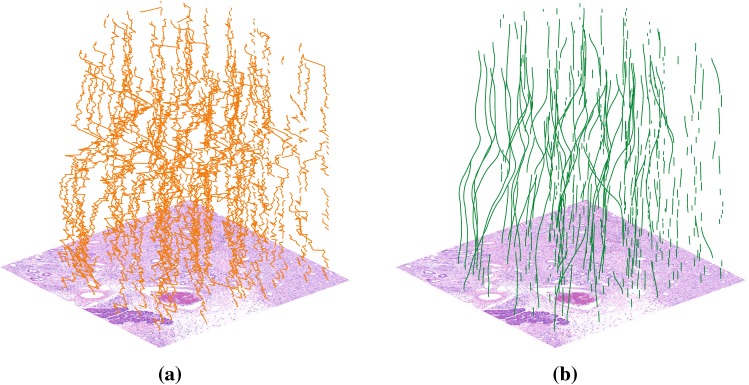
Real trajectories generated from a selected image portion: **a** original trajectories ${{\mathcal {T}}^j}$ before the registration and **b** final trajectories ${\mathcal {T}}_{\mathcal {Q}}^j$ after the smoothing process. Segmented trajectories due to discarded landmarks can be clearly observed

**Fig. 3:**
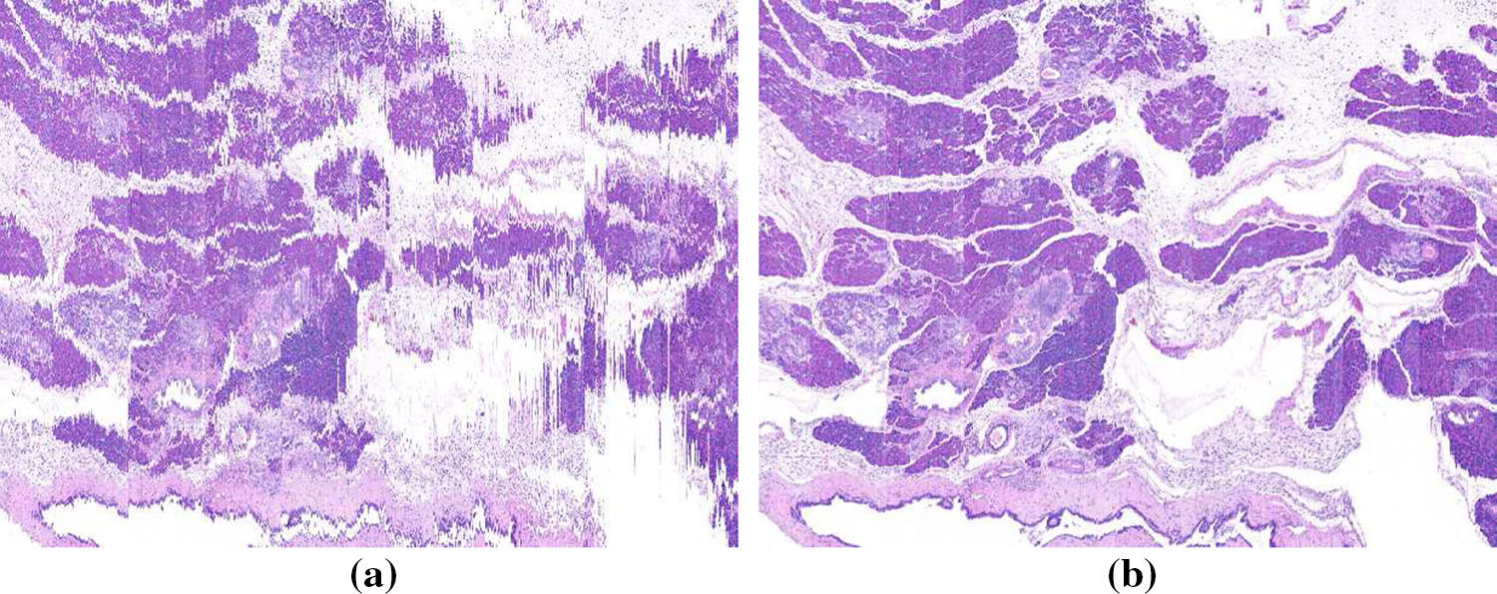
Reconstruction cross sections of a portion of 610 HE-stained images **a** before (only rigidly registered) and **b** after the proposed nonlinear registration

In order to accurately handle large deformations and reduce processing time, the aforementioned process is iterated with increasingly larger image resolutions and smaller template sizes $D \leftarrow \gamma D$, where $0< \gamma < 1$, in a coarse-to-fine multiscale fashion. The template search and landmark sampling areas are also updated accordingly.

The concept of smoothing trajectories of landmarks for 3D reconstruction has been previously proposed. For instance, Tan et al. [22] fitted smooth curves to the trajectories defined by a small set of points selected from the contours of the tissue, in order to optimize the affine transformations applied to the individual images. However, the method does not allow for nonlinear deformations inside the specimen. Gaffling et al. [7] manually selected landmarks along anatomical structures, using the respective smoothed trajectories as constrains to an intensity-based non-rigid registration. The sampling strategy and the complexity of the registration algorithm make this approach impractical for large datasets.

## Smoothness and isotropy evaluation

Several methods are available for evaluating the quality of the obtained reconstruction [6, 17]. However, for large datasets in which no gold standard is available, most of these methods cannot be used. Visual assessment, even when performed by experts, is subjective and provides no quantity measure, while methods that rely on manual delineations over the images are impractical for large amounts of images. Landmark-based validation is based on the distance between landmarks extracted from neighbor images, which, as previously stated, might not indicate a good reconstruction in the case of non-vertical anatomical structures.

Texture features are a viable approach for assessing the quality of a 3D reconstruction [5]. The method is based on gray-level co-occurrence matrix [9], which contains the frequency of pairs of voxels of certain intensities along a given direction. These frequencies are organized in a normalized array $g{\left( {i,j} \right) _{d,\alpha }}$, where *d* is the distance between pairs of voxels and $\alpha$ is the direction in which the pairs are evaluated. Thus, a contrast feature descriptor ${c_\alpha }$ can be defined as:

3 $$\begin{aligned} {c_\alpha } = \sum \limits _{i - j = 0}^{G - 1} {{{\left( {i - j} \right) }^2}\left( {\sum \limits _{i = 1}^G {\sum \limits _{j = 1}^G {g{{\left( {i,j} \right) }_{1,\alpha }}} } } \right) } \end{aligned}$$

where *G* is the number of distinct gray levels. Equation (3) can be used as a smoothness measure by averaging ${c_\alpha }$ over all cross sections along the direction $\alpha$.

A consistent reconstruction should not only be smooth, but also isotropic, presenting the same properties along any given direction. Assuming the statistical isotropy of the microanatomical structures observed in the microscopic images, the smoothness measure should be independent from the direction of the cross sections along which it is calculated. Thus, a necessary condition for isotropy is that ${{{\bar{c}}}_{{y_1}{y_2}}} \approx {{{\bar{c}}}_{{y_1}{y_3}}} \approx {{{\bar{c}}}_{{y_2}{y_3}}}$, where ${{\mathbf {y}}} = {\left( {{y_1},{y_2},{y_3}} \right) ^{\text {T}}}$ corresponds to the coordinates of the nonlinearly registered image set.

In the case of multiple stains, however, the contrast feature metric cannot be directly applied. The transitions between the stains in a given cross section would create high contrast values that do not represent the smoothness of the reconstruction. The method proposed here consists of binarizing image portions containing ducts and other structures, using different thresholds of hue, saturation and value for different stains. These structures appear as white holes on the images, regardless of the used stain. If the image is correctly reconstructed, these holes will form smooth 3D structures. Thus, a smoothness metric can be applied to the resulting binary image and averaged over the cross sections along a given direction.

This paper uses the average smoothness along different directions in order to compare the nonlinear reconstruction results with the rigidly aligned original images, as well as to verify that the smoothness of cross sections across slices is consistent with the smoothness of the slices themselves.

If no gold standard is available, when applied to methods based on the degree of matching between images or landmark coordinates, smoothness analysis alone cannot differentiate between a properly reconstructed image and a reconstruction presenting the “banana” effect [15]. However, as the proposed method’s image warping strategy retrieves the spatial connectivity of the anatomical structures, smoothness and isotropy are appropriate criteria for the accuracy evaluation of the reconstruction [20].

## Experiments

Experiments were conducted using a KPC mouse image dataset. This dataset consists of around 2500 images of $100\,\hbox {k}\times 60\,\hbox {k}$ pixels, scanned from 4-$\upmu$m sections of the pancreatic tumor of a KPC mouse model [23]. The experiments in this paper used a $15\,\hbox {k}\times 10\,\hbox {k}$ pixels downsampled version of the images. The images are grouped into five blocks (from 5-mm sections of the original specimen), each of which having the respective images stained with Hematoxylin & Eosin (HE) and Antigen KI-67 (Ki67) stains, and one block also stained with Cytokeratin-19 (CK19) and Masson’s Trichrome (MT) stains, in the following pattern: HE-Ki67-HE-CK19-HE-MT. In all experiments, the rigid registration used a template size of $1250\times 1250$ pixels and a search area of $2500\times 2500$ pixels. Two iterations of the non-rigid registration process were performed, with a starting template size *D* of $100\times 100$ pixels, a reduction rate $\gamma$ of 0.5 and a landmark sampling area set equal to *D*. The thresholds *T* and ${\theta _C}$ were set to, respectively, 0.1 and 0.3, while ${\theta _D}$ was also kept equal to *D*. The trade-off parameter $\lambda$ was set to $50^{-1}$. These parameters are generally noncritical and were empirically determined.

**Fig. 4:**
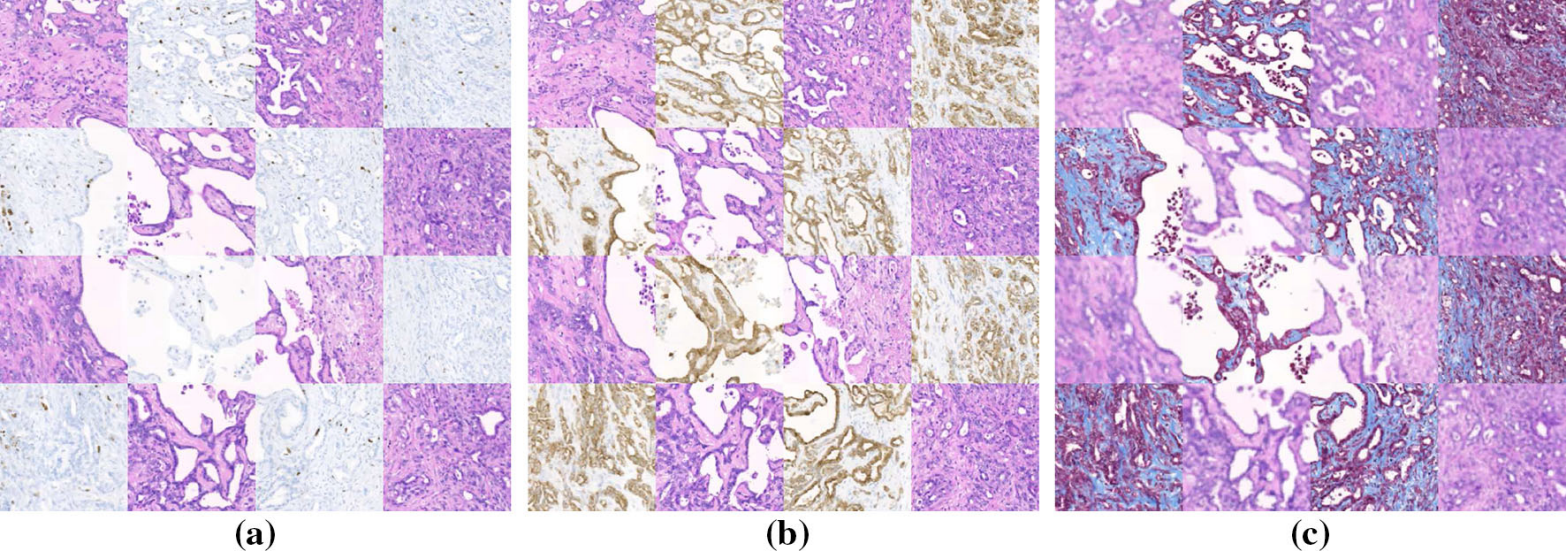
Registration results of image portions with multiple stains: **a** HE and Ki67, **b** HE and CK19, and **c** HE and MT

**Fig. 5:**
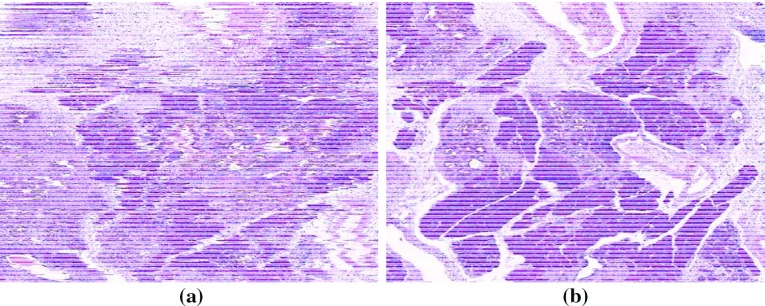
Reconstruction cross sections of a portion of 385 images of multiple stains **a** before (only rigidly registered) and **b** after the proposed nonlinear registration

**Fig. 6:**
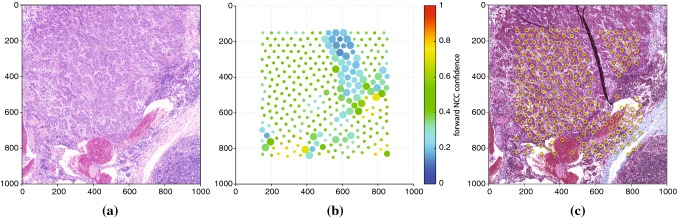
Example of landmark detection and artifact handling: **a** HE source portion, **b** detected landmark candidates, in which the color indicates the forward NCC confidence, and the radius corresponds to the backward template matching distance, and **c** final landmarks plotted over MT corresponding target portion, including a wrinkle. For illustrative purposes, the landmark sets shown here are denser than the sets used in the actual reconstructions

**Fig. 7:**
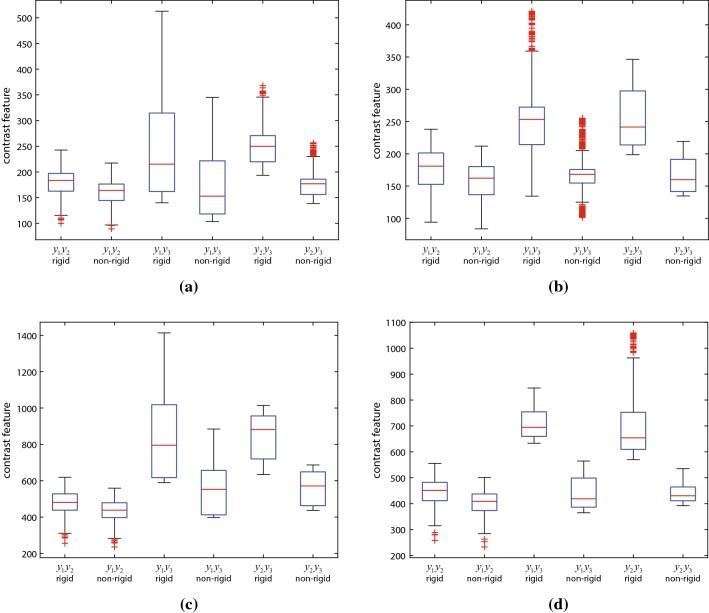
Smoothness isotropy analysis of four HE-stained image portions: **a**, **b** necrosis portions and **c**, **d** peripheral portions

**Fig. 8:**
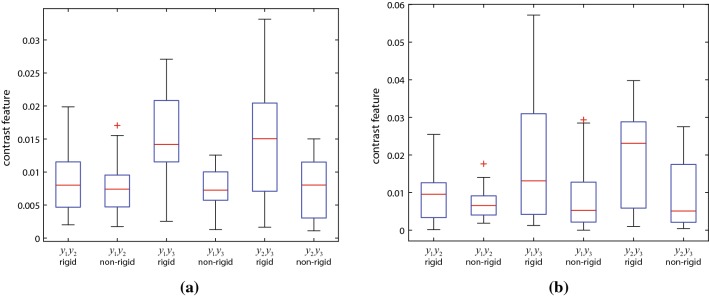
Smoothness isotropy analysis of two image portions with multiple stains

Figure 2a, b show, respectively, actual trajectories ${{\mathcal {T}}^j}$ and ${\mathcal {T}}_{\mathcal {Q}}^j$ for a selected image portion. The cross sections of an HE-stained image block, before and after the registration, are shown in Fig. 3. Several microstructures, such as ducts and blood vessels, virtually indistinguishable in Fig. 3a, can be clearly observed in Fig. 3b.

Figure 4 shows the results of the registration between adjacent images of different stains, while Fig. 5 shows cross sections from before and after the registration for an image block with multiple alternated stains. Again, high-contrast structures not visible in Fig. 5a can be clearly observed in Fig. 5b. In this reconstruction, the two iterations of the nonlinear registration generated an average of 4665 and 17,144 landmarks per image, respectively.

The process for detecting artifacts is shown in Fig. 6, where the HE image portion shown in Fig. 6a is registered with an adjacent MT portion containing a wrinkle, shown in Fig. 6c. The candidate landmarks selected using Eq. (1) are shown in Fig. 6b, in which the color indicates the forward NCC confidence, and the radius corresponds to the backward template matching distance. Figure 6c shows the final landmarks plotted over the target image, in which landmarks over the wrinkle region were eliminated by thresholds ${\theta _C}$ and ${\theta _D}$. For illustrative purposes, the landmark sets shown in Fig. 6 are denser than the sets used in the actual reconstructions.

In order to quantitatively evaluate the efficiency of the proposed registration method, the smoothness over three orthogonal directions was evaluated for ${\tilde{R}}\left( \cdot \right)$ and $R\left( \cdot \right)$. Two different experiments were performed. At first, four different cubic portions of side length 2.48 mm were selected from an HE-stained image block, two from the necrosis area of the tumor and two from the peripheral area. The average contrast was calculated for the cross section along ${y_1}$, ${y_2}$ and ${y_3}$ directions, with the ${y_3}$ direction (${y_1}{y_2}$ plane) corresponding to the original stacked images. The images were converted to gray scale and *G* set to 256.

The contrast results for the two necrosed areas are shown in Fig. 7a, b, while Fig. 7c, d display the contrast results for the peripheral areas. The ${y_1}{y_2}$ plane corresponds to the stacked images, in which contrast should be similar in both rigid and non-rigid cases. The rigidly registered images present much larger contrast values for the ${y_1}{y_3}$ and ${y_2}{y_3}$ planes. The non-rigid registration significantly reduces the average contrast value, which become very close to the values of the ${y_1}{y_2}$ plane. The contrast value for necrosed portions is lower than the peripheral portions due to the uniform pixel value in these areas.

The second experiment used two portions of side length 1.95 mm from a multi-stained image block, selected from areas with large number of ducts and blood vessels. The images where then binarized with independent thresholds of hue, saturation and value for each type of stain. The same thresholds were used for all images of a given stain and, as the stains were quite consistent along the slices, no intensities standardization was necessary. After the binarization, the contrast value (with $G = 2$) was calculated in a similar way to the previous experiment.

Figure 8 shows the results for the multiple stained portions. Due to the simple thresholding method used in the experiments, the resulting binary images are significantly noisy, resulting in smoothness values that do not correspond to perfect isotropy in neither direction. Nevertheless, these results confirm the efficiency of the proposed method in reconstructing images with multiple alternating stains.

## Conclusions

This paper proposes a landmark-based nonlinear 3D reconstruction method for histopathological images with multiple stains. The method directly optimizes the smoothness of trajectories defined by landmarks, detected along internal structures on the tissue, deforming all images simultaneously. This approach preserves the shape of these anatomical structures, avoiding the unnatural vertical structures often produced by pairwise registration methods.

The use of landmark-based registration, along with the relatively computationally simple template matching NCC function, allows the proposed method to be applied to the reconstruction of large pathological 3D images. Even though NMI is widely used for registering images from different modalities and could also be employed to find pairs of landmarks between images of different stains, its computational cost is much higher than that of NCC. Large datasets of images from the pancreas of a KPC mouse with both single and multiple stained images were successfully reconstructed, showing the efficiency of the NCC on matching corresponding points across roughly registered images with multiple alternating stains, which was confirmed by the smoothness analysis over binarized images. The method also automatically handles artifacts, which are unavoidable in the mounting and scanning of the slides and can deteriorate the registration process.

Although the reconstruction method minimizes the total variation in trajectories defined by the detected landmarks, it does not directly optimizes the isotropy of the reconstruction; nevertheless, the quantitative smoothness analysis demonstrates that the obtained reconstructions are nearly isotropic.

Current work includes the reconstruction of a new KPC mouse image dataset with more types of stains, which will allow the detection of different types of microstructures related to the dynamics of the tumor growth. Moreover, the proposed method will be applied on the reconstruction of the whole pancreatic tumors using the full-resolution images of the KPC mouse datasets.
